# Sarcopenia and Cardiogeriatrics: The Links Between Skeletal Muscle Decline and Cardiovascular Aging

**DOI:** 10.3390/nu17020282

**Published:** 2025-01-14

**Authors:** Dimitrios Anagnostou, Nikolaos Theodorakis, Christos Hitas, Magdalini Kreouzi, Ioannis Pantos, Georgia Vamvakou, Maria Nikolaou

**Affiliations:** 1 Department of Cardiology & 65+ Geriatric Outpatient Clinic, Amalia Fleming General Hospital, 14, 25th Martiou Str., 15127 Melissia, Greece; jimdimitris100@gmail.com (D.A.); n.theodorakis@flemig-hospital.gr (N.T.); ch.chitas@flemig-hospital.gr (C.H.); gvamva@yahoo.gr (G.V.); 2 School of Medicine, National and Kapodistrian University of Athens, 75 Mikras Asias, 11527 Athens, Greece; 3 NT-CardioMetabolics, Clinic for Metabolism and Athletic Performance, 47 Tirteou Str., 17564 Palaio Faliro, Greece; 4 Department of Internal Medicine & 65+ Geriatric Outpatient Clinic, Amalia Fleming General Hospital, 14, 25th Martiou Str., 15127 Melissia, Greece; kreouzi.m@live.unic.ac.cy; 5 Department of Radiology, Amalia Fleming General Hospital, 14, 25th Martiou Str., 15127 Melissia, Greece; ipantos@gmail.com

**Keywords:** sarcopenia, cachexia, cardiogeriatrics, elderly, lean mass, muscle mass, aging, metabolism, physiology, cardiovascular disease, heart failure

## Abstract

Sarcopenia, an age-related decline in skeletal muscle mass, strength, and function, is increasingly recognized as a significant condition in the aging population, particularly among those with cardiovascular diseases (CVD). This review provides a comprehensive synthesis of the interplay between sarcopenia and cardiogeriatrics, emphasizing shared mechanisms such as chronic low-grade inflammation (inflammaging), hormonal dysregulation, oxidative stress, and physical inactivity. Despite advancements in diagnostic frameworks, such as the EWGSOP2 and AWGS definitions, variability in criteria and assessment methods continues to challenge standardization. Key diagnostic tools include dual-energy X-ray absorptiometry (DXA) and bioimpedance analysis (BIA) for muscle mass, alongside functional measures such as grip strength and gait speed. The review highlights the bidirectional relationship between sarcopenia and cardiovascular conditions such as heart failure, aortic stenosis, and atherosclerotic cardiovascular disease, which exacerbate each other through complex pathophysiological mechanisms. Emerging therapeutic strategies targeting the mTOR pathway, NAD+ metabolism, and senescence-related processes offer promise in mitigating sarcopenia’s progression. Additionally, integrated interventions combining resistance training, nutritional optimization, and novel anti-aging therapies hold significant potential for improving outcomes. This paper underscores critical gaps in the evidence, including the need for longitudinal studies to establish causality and the validation of advanced therapeutic approaches in clinical settings. Future research should leverage multi-omics technologies and machine learning to identify biomarkers and personalize interventions. Addressing these challenges is essential to reducing sarcopenia’s burden and enhancing the quality of life for elderly individuals with comorbid cardiovascular conditions. This synthesis aims to guide future research and promote effective, individualized management strategies.

## 1. Introduction

Sarcopenia refers to the age-related decline in skeletal muscle mass and function. An effort to define sarcopenia has been undertaken by at least eight different consensus groups over the last 15 years, resulting in a constantly evolving definition with no universally accepted consensus [1]. While efforts to reach a global consensus are underway [2], almost all available definitions to date encompass three components: (1) a decrease in muscle mass, (2) a decline in muscle strength, and (3) evidence of impaired muscle physical function [1].

Sarcopenia is associated with a wide range of adverse outcomes, including frailty, loss of independence, increased risk of falls and fractures, and diminished quality of life. Of particular significance is the growing body of evidence linking sarcopenia to cardiovascular diseases (CVD), including heart failure, aortic stenosis, atherosclerotic cardiovascular disease, peripheral arterial disease, and atrial fibrillation. The interplay between sarcopenia and CVD is multifaceted, involving shared mechanisms such as chronic inflammation (inflammaging), hormonal dysregulation, oxidative stress, and physical inactivity, which exacerbate both conditions.

This review aims to provide an up-to-date synthesis of the current state-of-the-art regarding sarcopenia in the context of cardiogeriatrics. It will explore the pathophysiological underpinnings, diagnostic challenges, and therapeutic approaches, highlighting gaps in evidence and potential directions for future research. The goal is to advance understanding and promote strategies to mitigate the burden of sarcopenia in aging populations, particularly those with comorbid cardiovascular conditions.

Systematic reviews and meta-analyses have previously examined the topic of sarcopenia, investigating prevalence rates in cardiovascular conditions [3,4], the effects of interventions such as resistance training [5], and the therapeutic potential of pharmacological agents and nutritional supplementation [6]. However, most of these studies naturally have a narrower focus, often attempting to answer very specific research questions. Notably, many meta-analyses report substantial heterogeneity, likely due to the evolving and numerous definitions of sarcopenia. Furthermore, in many cardiovascular conditions, the scarcity of data limits the feasibility of conducting such studies, but cohort or case-control studies do exist. In this paper, a broader approach was adopted, aiming to synthesize the available evidence across a wide spectrum of cardiovascular diseases, including pertinent pathophysiologic links where possible, even in areas where quantitative synthesis has not yet been feasible.

## 2. Materials and Methods

A literature review was conducted to identify studies published up to 10 December 2024. The search was performed using electronic databases, including PubMed, Embase, and Scopus. The search strategy incorporated both free-text terms and controlled vocabulary, such as Medical Subject Headings (MeSH), to ensure thorough coverage of the relevant literature. Boolean operators (AND, OR) were applied to combine search terms and refine the search process. Key terms included “sarcopenia”, “cachexia”, “cardiogeriatrics”, “heart failure”, “aortic stenosis”, “transcatheter aortic valve implantation”, “atrial fibrillation”, “peripheral arterial disease”, “inflammaging”, “metainflammation”, “muscle mass”, “lean mass”, “aging”, “mTOR”, and “cardiovascular disease”. The searches were not restricted by publication type but were limited to studies written in English. Pertaining to sarcopenia definitions, relevant consensus documents were reviewed and summarized. Regarding the pathophysiologic mechanisms behind sarcopenia, relevant review articles containing widely accepted mechanisms or mechanisms of potential clinical interest were included at the authors’ discretion. While incorporating studies addressing the intersection of sarcopenia with cardiogeriatric conditions, an evidence hierarchy was followed. Priority was given to systematic reviews and meta-analyses, followed by randomized controlled trials (RCTs), cohort studies, and, lastly, case-control studies.

## 3. Diagnosis of Sarcopenia and Cachexia

Muscle mass is typically approximated using an imaging technique such as dual-energy X-ray absorptiometry (DXA), which measures lean body mass, or via bioimpedance analysis (BIA), which estimates muscle mass based on the body’s electrical conductivity.

Muscle strength is usually assessed by measuring grip strength using a dynamometer. Information about muscle physical function is derived through the assessment of complex, multifactorial processes, with measures such as gait speed reflecting the integration of muscle strength, coordination, balance, and cardiorespiratory capacity.

Two widely utilized definitions of sarcopenia are those established by the Asian Working Group for Sarcopenia (AWGS), updated in 2019, and the European Working Group on Sarcopenia in Older People (EWGSOP2), revised in 2019 (Table 1).

According to the EWGSOP2 consensus, sarcopenia can also be classified as primary (age-related) when no specific underlying cause is identified, and as secondary when associated with identifiable factors, such as systemic illnesses (e.g., cancer, heart failure, renal failure), nutritional deficiencies (e.g., inadequate caloric intake), or specific conditions and circumstances (e.g., disability or muscle disuse). When sarcopenia coexists with obesity, a diagnosis of sarcopenic obesity is supported, despite the lack of globally adopted criteria [7]. Sarcopenia can also coexist with decreased bone mineral density, a condition termed either sarco-osteopenia (when osteopenia coexists), or sarco-osteoporosis (when osteoporosis coexists) [8].

Cachexia is a close but distinct term that refers to a wasting syndrome characterized predominantly by significant, edema-free, unintentional weight loss in the context of chronic diseases such as cancer or heart failure. This condition also involves the loss of skeletal muscle mass, with or without concurrent loss of fat tissue, and can therefore present as (secondary) sarcopenia. However, supporting a diagnosis of cachexia typically requires additional criteria, including clinical features such as anorexia, fatigue, and specific biochemical abnormalities. These include anemia, hypoalbuminemia, and elevated inflammatory markers such as C-reactive protein (CRP) and interleukin-6 (IL-6) [9]. Histological differences between cases of cachexia and sarcopenia also exist. As will be discussed, in the specific case of cachexia due to heart failure, a muscle fiber shift towards a type II fiber predominance is noted, while in sarcopenia, a shift towards a type I fiber predominance is observed [10].

## 4. Pathophysiology of Sarcopenia

**Figure 1:**
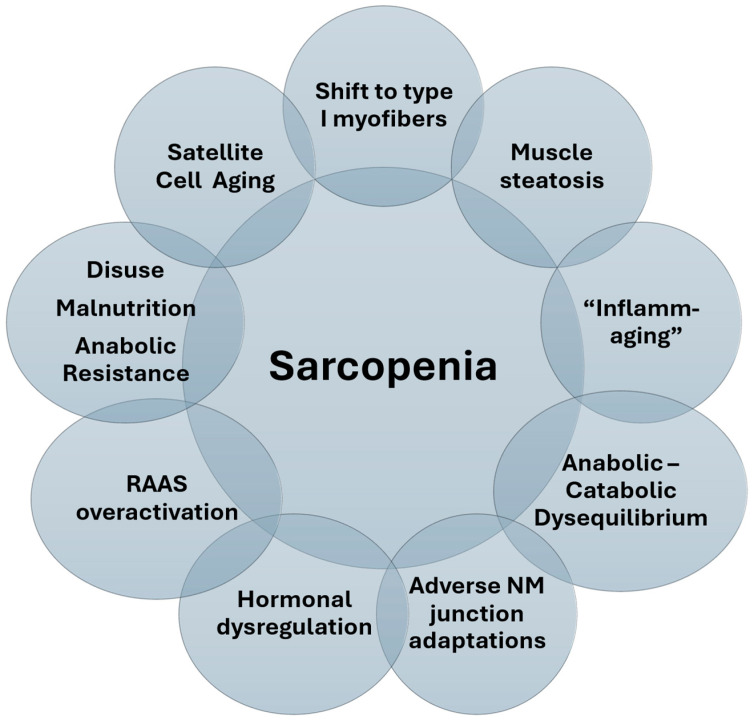
Mechanisms that have received research interest and are implicated in the pathophysiology of sarcopenia. Abbreviations: RAAS: Renin Angiotensin Aldosterone System, NM: Neuromuscular (junction).

An in-depth and extensive description of the molecular and pathologic processes that lead to sarcopenia development is outside of the scope of this review. However, a few key processes well supported in literature or of potential interest to clinicians are mentioned (see also Figure 1).

### 4.1. Diminished Muscle Regenerative Capacity-Satellite Cell Aging and Geroconversion

Muscle satellite cells are somatic (adult) stem cells involved in muscle maintenance, regeneration, and response to injury [11]. They are located beneath the basal lamina of skeletal muscle fibers. In their resting (quiescent) state, they express the transcription factor Pax7. Upon activation, such as in response to muscle injury, satellite cells retain Pax7 expression and express myogenic regulatory factors (MRFs), such as Myf5, MyoD, MyoG, and Mrf4 [12]. Activated satellite cells can undergo asymmetric division, with one daughter cell maintaining the satellite cell pool and the other differentiating into a myogenic precursor cell. These precursor cells (myoblasts) contribute to muscle repair by fusing with existing myocytes. During aging, there is a decline in satellite cell number and function, leading to diminished muscle regenerative capacity [13]. Intrinsic factors, including genomic instability, epigenetic changes, mitochondrial dysfunction, and impaired autophagy, contribute to reduced proliferative capacity and diminished myogenic potential of satellite cells [13]. Extrinsic factors, including alterations in the satellite cell niche, chronic inflammation, disrupted TGF-β and Notch signaling pathways, and reduced availability of “rejuvenating” systemic factors such as GDF11, may also promote satellite cell dysfunction [13,14]. Geroconversion, a process where aged satellite cells transition from a quiescent state to a pre-senescent or fully senescent state upon regenerative pressure, impairing their ability to sustain muscle repair has been demonstrated in mice [15]. Together, these factors compromise muscle regeneration and may contribute to the development of sarcopenia.

### 4.2. Decrease in Muscle Quality-Shift Towards Type I Fibers and Fatty Infiltration

It is well-supported that during aging, skeletal muscle undergoes significant changes in fiber composition and quantity, characterized by an early, preferential loss of Type II (fast-twitch) fibers and, therefore, a relative increase in the proportion of Type I (slow-twitch) fibers. As aging progresses, the absolute numbers of both types of fiber decrease [16,17,18,19,20].

In addition, a hallmark of skeletal muscle aging is fat infiltration (myosteatosis). Fat exists in adipocytes between different muscle groups (intermuscular fat) and between myofibers of the same group (intramuscular fat). Finally, there is a smaller lipid pool inside muscle cells themselves (intramyocellular lipids) [21]. Mechanisms that lead to myosteatosis may involve chronic low-grade inflammation, mitochondrial dysfunction, and impaired lipid metabolism [22]. Whether these processes are strictly due to aging, or whether they are secondary to illness, altered nutritional intake, or disuse, is not definitively proven [22,23]. Myosteatosis is linked to insulin resistance and thus may be particularly important in the pathogenesis of sarcopenic obesity [24]. Whether as primary drivers or simply as markers of aging muscle, myosteatosis, along with altered myofiber content, reflect decreased muscle quality and may contribute to sarcopenia development.

**Figure 2:**
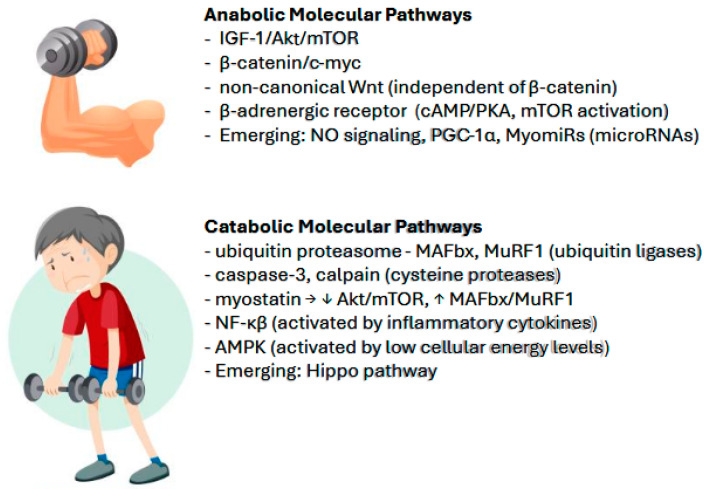
An in-depth understanding of sarcopenia involves the elucidation of key anabolic and catabolic molecular pathways in adult skeletal muscle. This understanding provides insight into potential therapeutic targets and guides future research. Potential links with cardio-geriatric conditions also emerge as in the case of inflammation—a hallmark of many cardiovascular disease states—activating catabolism through NF-κ pathway. ↓ (decreased); ↑ (increased).

The key anabolic and catabolic molecular pathways associated with the development and progression of sarcopenia are illustrated in Figure 2.

### 4.3. Molecular Regulation of Muscle Anabolism and Catabolism-Dysequilibrium Favors Sarcopenia

Muscle is a dynamic tissue that constantly undergoes remodeling through a balance between anabolic (protein synthesis) and catabolic processes (protein degradation). This equilibrium is crucial for maintaining muscle mass and function, but its disruption, favoring catabolism, may lead to sarcopenia [25]. Therefore, an understanding of the underlying molecular pathways and signal cascades for both processes (anabolic and catabolic) is important.

On a molecular level, the best-described anabolic pathway involves the insulin-like growth factor 1 (IGF-1)/Akt/mTOR signaling cascade, which promotes protein synthesis and muscle growth upon stimulation with various factors, including nutrients (amino acids), growth factors (IGF-1, insulin), and mechanical strain [26]. Other anabolic pathways involve the β-catenin/*c*-myc signal cascade, a “non-canonical” Wnt pathway (independent of β-catenin), and the β-adrenergic receptor signaling pathway. The latter (β-receptor) is G-protein-coupled and triggers adenylyl cyclase/cAMP/PKA cascade signaling upon stimulation [27]. It also activates mTOR through a different mode of action, and both pathways seem to contribute to its muscle-sparing effects [27]. Experiments have shown that β-receptor stimulation leads to skeletal muscle hypertrophy in both animals and humans, albeit not always functional or resulting in increased force production [27]. Finally, emerging anabolic pathways under research include the nitric oxide (NO) signaling pathway and the peroxisome proliferator-activated receptor gamma-coactivator 1 alpha (PGC-1α) pathway. A muscle-specific family of micro RNAs termed myomiRs is also under study [27].

Regarding catabolism, one important catabolic pathway involves the ubiquitin-proteasome pathway (UPP). It is known that proteins tagged with the protein ubiquitin are flagged for degradation by the proteasome, a large protein complex [28]. This results in protein degradation and facilitates protein recycling. Two key genes involved in the muscle catabolism process are the muscle-specific F-box protein gene (MAFbx) and the muscle ring finger-1 (MuRF1) genes, which specifically encode ubiquitin ligases [27]. Indeed, their inactivation impairs muscle atrophy [27]. Muscle hypertrophy is also affected, as protein degradation and turnover are essential for both muscle hypertrophy as well as maintenance of muscle mass [29]. Additionally, it is known that MAFbx/MuRF1 genes are activated by a specific class of transcription factors called FOrhead BoX O (FOXO) [27]. Evidence for this stems from the fact that Akt, a component of the anabolic IGF-1/Akt/mTOR cascade, can phosphorylate FOXO factors, thereby preventing the activation of MAFbx/MuRF1 genes and inhibiting catabolism [27]. This reflects cross-talk between anabolic and catabolic pathways. In addition, one isoform of FOXO (FOXO3) seems to control autophagy in skeletal muscles, a process that also contributes to protein degradation and turnover [30]. Proteolytic systems in skeletal muscle also include caspase-3 and calpain systems [31]. Caspase-3 and calpain are cysteine proteases. Caspase-3 is activated by apoptotic signals via caspase cascades. Calpain activity increases by the increase in Ca2+ levels that happens during periods of muscle inactivity [31]. Both systems contribute to muscle atrophy through the cleavage of structural and regulatory proteins.

A different catabolic pathway involves myostatin. Myostatin (MSTN, or GDF-8) is a cytokine produced by skeletal muscle (myokine) and is part of the transforming growth factor-beta (TGF-β) superfamily [32]. An inactive form is initially produced by skeletal muscle cells and undergoes proteolytic cleavage to form active MSTN [32]. MSTN binds to activin receptor type IIB (ActRIIB), which activates activin receptor-like kinase 4 (ALK4) or ALK5 receptors and triggers intracellular signaling via phosphorylation of SMAD proteins. Possibly through SMAD3 mediation, the activity of the anabolic IGF-1/Akt/mTOR pathway is inhibited [33]. In addition, SMAD2/3, after forming a complex with SMAD4, gets translocated to the nucleus and upregulates the expression of MAFbx and MuRF1 genes [32]. Non-SMAD pathways with the same endpoint have also been described [32]. Through these mechanisms, MSTN inhibits muscle anabolism and promotes muscle catabolism.

Apart from the above, a distinct catabolic molecular cascade involves AMP-activated protein kinase (AMPK). AMPK functions as a molecular “energy sensor” that is activated when cellular energy levels are low. AMPK inhibits anabolic IGF-1/Akt/mTOR signaling while promoting catabolic signaling through the expression of FOXO transcription factors [27]. Similarly, another signaling cascade that promotes muscle atrophy involves nuclear factor κB (NF-κB), which is a transcription factor. NF-κB signaling is characteristically activated by inflammatory cytokines of the TNF family, such as TNF-α and TWEAK (TNF-like weak inducer of apoptosis) cytokines, leading to MuRF1 upregulation and muscle atrophy [27]. This highlights an important molecular link between inflammation and muscle catabolism. Lastly, the more recently found hippo pathway, though repression of YAP/TAZ signaling and suppression of TEAD transcription factors, also promotes muscle catabolism [27].

### 4.4. Hormonal Contributary Factors

During aging, various hormonal changes may contribute to the development of sarcopenia. Growth hormone (GH, or somatotropin), secreted from the anterior pituitary gland in response to growth hormone-releasing hormone (GHRH) stimulation, promotes the production of insulin-like growth factor 1 (IGF-1) primarily by the liver. IGF-1, through the IGF-1/Akt/mTOR signal cascade, promotes an anabolic muscle state [26]. During aging, GH and IGF-1 levels decrease in part due to a reduction in GHRH release from the hypothalamus [29]. Ghrelin, a neuropeptide released from the GI tract in response to hunger, and which promotes GH secretion, may also be implicated [34]. The role of sex hormones (testosterone and estrogen) also remains to be elucidated. Testosterone, along with its biologically more active form, dihydrotestosterone (DHT), acts via binding to the androgen receptor (AR) in the cytoplasm [35]. After this, the AR-testosterone/DHT complex translocates to the nucleus and binds androgen response elements (AREs) to regulate the transcription of various genes. The overall process promotes protein synthesis and an anabolic muscle state [35]. Testosterone levels decrease with age, and testosterone administration in males with hypogonadism has been associated with increases in muscle mass, but the evidence is not conclusive [36]. The role of estrogen is less well-studied, but it is thought to be mediated by estradiol receptors (ER), which play a key role and contribute to muscle health [37,38]. Estradiol is the biologically potent form of estrogen, and its levels decline after menopause [38]. Post-menopausal estrogen replacement has yielded conflicting results for muscle strength and size [37]. Cortisol, a catabolic hormone, has also attracted research interest and may be involved in muscle mass reduction during aging [37]. Finally, there seems to exist a bidirectional relationship between aged sarcopenic muscle and insulin sensitivity. Specifically, sarcopenia promotes insulin resistance and insulin resistance leads to muscle catabolism and fuels sarcopenia [37]. Other substances including adipokines (leptin and adiponectin), vitamin D, and thyroid hormones have also been suggested as playing a role in the pathogenesis of sarcopenia [34,35,36,37].

### 4.5. Role of Inflammation—“Inflamm-Aging”, “Garb-Aging” and “Meta-Inflammation”

Chronic low-grade systemic inflammation, often seen with aging, is thought to promote muscle atrophy [34,35]. In fact, levels of specific inflammatory substances, including TNF-α, IL-6, and C-reactive protein (CRP), have shown an inverse correlation with muscle mass and strength [25]. As already mentioned, through NF-κB signaling, inflammatory cytokines of the TNF family lead to muscle atrophy. Extending the above concept, the accumulation of different kinds of molecular “debris” in the form of pathogen-associated molecular patterns (PAMPs), damage-associated molecular patterns (DAMPs), nutritional products, or products from gut microbiota can fuel this auto-inflammatory process (“garb-aging”) [39]. “Metainflammation” (metabolic inflammation) refers to inflammation accompanying an excess nutrient state, has been linked to insulin resistance, and may also provide an additional mechanism that sustains the low-level chronic inflammatory state seen with aging [40,41,42]. After all, it is known that myocyte fat infiltration (characteristic of sarcopenic muscle) is associated with mitochondrial dysfunction and reactive oxygen species (ROS) production (lipotoxicity) [25].

### 4.6. Role of Renin Angiotensin Aldosterone System (RAAS)

Interestingly, the renin-angiotensin-aldosterone system (RAAS) may also be implicated in the pathogenesis of sarcopenia [43]. Indeed, animal studies have linked increased levels of ATII with decreased muscle strength [44]. On a molecular basis, ATII decreases the activity of the anabolic Akt/mTOR pathway while promoting muscle degradation via the ubiquitin-proteasome and caspase-3 systems [37]. ATII blockage with angiotensin receptor blockers has shown muscle protective effects in a small observational study of dialysis patients [45]. Data from animal studies also hint at this [37]. Further research would be useful given the widespread use of RAAS-blocking agents in many cardiovascular and renal disease states.

### 4.7. Roles of Neuromuscular Junction, Effects of Disuse, and Nutrition

The neuromuscular junction displays many adverse adaptations in response to aging that may contribute to, or be the result of, sarcopenia development, as recently reviewed by Deschenes et al. [46]. Disuse due to decreased physical activity, hospitalizations, or immobilization in older adults also accelerates sarcopenia development [47]. In addition, nutrition and specifically diminished protein intake are likely contributors to sarcopenia, as several prospective studies have linked decreased protein intake with decreased muscle mass and strength [48]. Highlighting the importance of muscle use and adequate nutrition is a phenomenon called “anabolic resistance”, which has been observed in aging muscle. Specifically, as age progresses, nutritional and exercise stimuli exert diminished anabolic effects on muscle, perhaps related to weaker Akt/mTOR pathway stimulation [49].

## 5. Sarcopenia and Cardiovascular Diseases of the Elderly

### 5.1. Heart Failure

Heart failure (HF) is a constellation of typical symptoms and/or signs of congestion accompanied by objective evidence of decreased ejection fraction (EF) or elevated left ventricular filling pressures [50]. The prevalence of HF rises with age, ranging from approximately 1% in individuals under 55 years to over 10% in those aged 70 years and older, making it an extremely relevant condition in the elderly population [50].

Two distinct archetypes of muscle involvement are observed in elderly patients with heart failure. The first is noted in cases of cachexia resulting from severe heart failure and the second in cases of sarcopenia coexisting with heart failure. In cardiac cachexia, a shift towards a type II (fast-twitch) myofiber type is noted, along with intrafibrillar edema, fat infiltration, and fibrosis, perhaps related to NT-pro-BNP secretion [10]. A hypermetabolic energy state with notable inflammation is also observed [10]. In sarcopenia, a type I (slow-twitch) fiber predominance, often with fat infiltration, is characteristic, and there is a hypometabolic or normometabolic energy state [10]. A distinction between the two conditions is often challenging, especially if they co-exist.

Furthermore, in the specific case of sarcopenia coexisting with HF, there is evidence for a bidirectional link between the two conditions. Firstly, sarcopenia can lead to decreased physical performance and altered ergoreflex and sympathetic nervous system modulation, contributing to HF pathogenesis [10]. Secondly, sarcopenia contributes to muscle fatigue, dyspnea, and diminished quality of life in patients with HF [10,51]. From the opposite end, a plethora of HF-related factors can contribute to sarcopenia pathogenesis and progression. These include the state of physical inactivity seen in HF, malnutrition, and malabsorption, increased oxidative stress, hormonal changes such as increased ATII levels, decline in GH/IGF-1 levels, and myostatin upregulation, as well as decreased muscle blood flow [10]. Moreover, as per a recent meta-analysis, low appendicular skeletal mass and the presence of sarcopenia have been linked to higher natriuretic peptide levels in patients with HF [52]. How low muscle mass, weight loss, and cachexia are all linked to higher BNP or NT-proBNP levels is not entirely clear [52].

From a clinical standpoint, according to a recent meta-analysis by Chen et al., the prevalence of sarcopenia in HF patients is about 31% versus 6–12% in the general population [53]. However, in this meta-analysis (as in previous similar efforts [4]), extreme heterogeneity between studies was noted (*I*^2^ 98%), perhaps related to the absence of universal criteria for sarcopenia definition. Most included studies defined sarcopenia using AWGS or EWSOP criteria. Interestingly, on subgroup analysis, the prevalence of sarcopenia was higher in those with reduced EF (28%) compared to those with preserved EF (18%), and more common in elderly patients aged 65 or more (31%) versus younger patients (25%) [53]. Male sex showed a slightly higher prevalence of sarcopenia as well (34% vs. 32% for females), but this was not consistent with the results of previous studies and, hence, should be interpreted with caution [53].

Lastly, the prevalence of sarcopenia in HF inpatients was higher (39%) compared to outpatients (20%) [53]. Perhaps more alarming were the associations with adverse clinical outcomes. Specifically, sarcopenia was associated with increased mortality (HR of 2.06) and hospitalizations (HR of 1.2) [53]. Notably, in a subgroup analysis, while the effect of sarcopenia in patients with HF and reduced EF was statistically significant for predicting poor prognosis, the effect in patients with HF and preserved EF was not [53]. More studies are needed before sarcopenia can be definitively linked to poor prognosis in HF patients with preserved EF. In addition, based on data from 200 patients enrolled in a SICA-HF trial, muscle wasting is also associated with a decline in cardiorespiratory fitness (CRF) in patients with HF, independent of factors such as age, sex, NYHA class, and hemoglobin level [51]. This could contribute to HF symptom development and decreased quality of life in those patients [54]. In the specific case of obese sarcopenic HF patients (sarcopenic obesity) with reduced EF, sarcopenia was associated with an additional reduction in cardiorespiratory fitness parameters (such as VO_2_ max), assessed via cardiopulmonary exercise testing [55]. This was independent of factors such as age, sex, HF biomarker status, and fat mass percentage. However, this was a small retrospective study of only 40 patients; hence, replication in larger studies is warranted.

**Figure 3:**
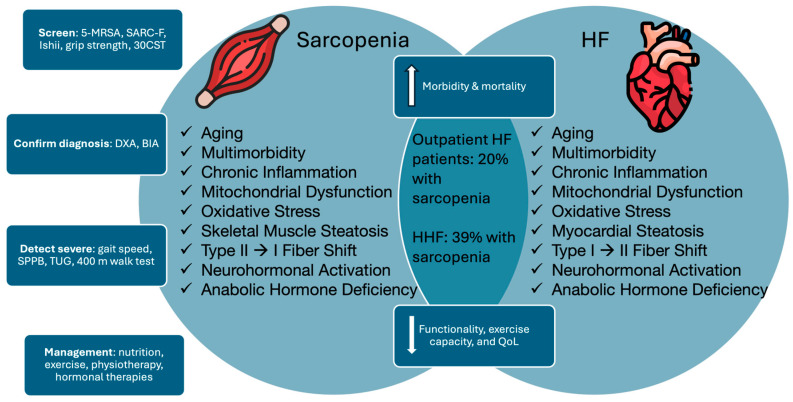
Screening for, diagnosing, and managing sarcopenia is crucial to prevent morbidity and mortality in patients with heart failure. Abbreviations-HF: Heart Failure, HHF: Hospitalized Heart Failure patients, SPPB: Short Physical Performance Battery, DXA: Dual-energy X-ray absorptiometry, BIA: Bioimpedance analysis, TUG: Time Up-and-Go, 30CST: 30 s sit-to-stand test, Ishii: Ishii score for sarcopenia, 5-MRSA: 5-question Mini Sarcopenia Risk Assessment questionnaire, QoL: Quality of Life.

The overlapping and interconnected pathophysiological mechanisms of HF and sarcopenia are illustrated in Figure 3.

### 5.2. Aortic Stenosis and tAVI

The prevalence of aortic stenosis (AS) in patients over 75 years of age is 12.4%, and severe AS is present in 3.4% of them [56]. Sarcopenia and AS could potentially implicate common pathophysiological mechanisms. Both conditions are age-related and may involve underlying chronic low-grade inflammation (“inflammaging”) [40,57,58]. A low output state secondary to AS, or RAAS system overactivation, may also promote muscle wasting [43]. Additionally, muscle wasting could lead to functional decline and cardiovascular deconditioning, exacerbating symptoms in patients with AS. Fortunately, contemporary transcatheter aortic valve implantation (TAVI) has revolutionized the management of AS, offering a less invasive alternative to surgical aortic valve replacement (SAVR), particularly for patients at high or prohibitive surgical risk. Most studies available on patients undergoing TAVI provide a loose definition of sarcopenia based solely on the reduction in skeletal muscle mass. With this in mind, 21–70.2% of patients undergoing TAVI have evidence of decreased muscle mass on CT assessment, as Bertschi et al. noted in their systematic review [59]. Using this imaging modality, the psoas muscle area, paravertebral muscle area, or total muscle area are usually assessed at the levels of the 3rd or 4th lumbar vertebrae (L3/L4). As the authors also noted, there was high heterogeneity between studies attributed, in part, to different muscles assessed and different cut-offs for defining “low muscle mass”. Pooling of data for the performance of a meta-analysis was not possible. Furthermore, CT-based diagnostic criteria for low muscle mass have not been standardized; for example, neither the EWGSOP2 nor the AWG definitions provide specific cutoffs [60,61]. However, a cautious examination of this review suggests that low muscle mass on its own might predict late (>1 year) mortality after TAVI might be linked to prolonged hospital length of stay and functional decline, although more studies are needed to prove this [59]. It is important to note that low muscle mass by itself requires cautious interpretation, as it could simply reflect an advanced “cachectic” disease state rather than true sarcopenia.

Perhaps more relevant is a study by Mamane et al. involving 400 elderly patients (mean age 83.5 ± 5.7 years) undergoing transcatheter aortic valve implantation (TAVI). In this study, sarcopenia was defined as a decrease in both muscle mass (assessed by CT) and muscle strength (assessed via chair-rise performance). The authors reported that 21% of the TAVI patients met the criteria for sarcopenia and further found that sarcopenia was associated with a hazard ratio of 11.30 (95% CI: 2.51–50.91) for predicting late (>1 year) mortality [62]. Sarcopenia was also associated with worsening disability and discharge to a skilled-care facility in this study [62].

In a more recent study by Stein et al. of 445 symptomatic patients with severe aortic stenosis undergoing TAVI, the authors examined three components of sarcopenia separately: muscle mass by CT, grip strength, and gait speed [63]. Of them, the psoas muscle area indexed to height, grip strength, and slow gait speed were predictive of all-cause mortality after TAVI, but only in unadjusted analysis. After adjustment, only slow gait speed retained statistical significance with an adjusted HR of 1.12 (95% CI 1.04–1.21) per 0.1 m/s decrease in gait speed. Per the EWSOP2 definition of sarcopenia, a reduction in walking speed, in addition to low muscle strength and low muscle mass, would be considered severe sarcopenia (Table 1) [60]. So far, no studies that we are aware of have assessed muscle mass in TAVI patients with DXA. A question remains whether a whole-body muscle assessment via this method, which also has better-defined consensus cutoffs, would lead to the same results and possibly lower heterogeneity. The importance of adopting standardized, consensus-based definitions of sarcopenia in future studies cannot be overstated. Finally, it is tempting to wonder whether reversing sarcopenia components with nutritional support or exercise interventions pre-TAVI could lead to a better patient prognosis. Hopefully, future trials will explore this possibility in greater depth.

### 5.3. Atherosclerotic Cardiovascular Disease

The term atherosclerotic cardiovascular disease (ASCVD) refers to a group of cardiovascular conditions caused by atherosclerotic plaque buildup. It includes conditions such as acute coronary syndromes (ACS), coronary artery disease (CAD), and peripheral arterial disease (PAD). Again, the concept of chronic low-level inflammation (“inflammaging”) could be a common mechanism underlying both sarcopenia and ASCVD, as well as an age-related component that exists for both conditions [57,58,64].

#### 5.3.1. Acute Coronary Syndromes (ACS)

A retrospective cohort study by Won et al. of 303 patients with ST-elevation myocardial infarction (STEMI) undergoing primary coronary intervention (PCI) showed that a ratio of serum creatinine to cystatin C (sarcopenia index, SI) less than 0.9 on admission, a surrogate for muscle mass per recent literature [65], was associated with an HR of 3.01 (95% CI: 1.22–7.38) for the occurrence of major adverse cardiovascular events (MACEs) at 1 year post-PCI [62]. Using a prospective study design, Lee et al. also examined the value of SI in 1086 elderly patients who underwent PCI. A SI value of 0.79 ± 0.15 was associated with an adjusted HR of 2.18 for predicting MACEs at the 3-year follow-up compared to an SI value of 1.13 ± 0.21 [66]. Another retrospective cohort study by Sato et al. of 387 STEMI patients who underwent muscle mass assessment via DXA associated low appendicular skeletal muscle mass index (ASMI) with all-cause mortality (adjusted HR 2.06, 95% CI 1.01–4.19) during the 33 months of follow-up [67]. Interestingly, when necessitating additional formal sarcopenia criteria, such as low muscle strength (as per AWGS sarcopenia definition), statistical significance was not achieved (by a thin margin) [67]. Finally, an observational prospective study by Matsumoto et al. of 132 patients with non-STEMI revealed that a psoas muscle index (PMI) less than 772 mm^2^/m^2^ assessed via CT was an independent predictor of future cardiovascular events (HR 3.3, 95% CI: 1.7–6.3) [68]. All the above studies provide a signal that low muscle mass (one component of sarcopenia) could be a marker of poor prognosis in patients with acute coronary syndromes. However, sarcopenia requires additional criteria for diagnosis relating to muscle strength or physical function. We cannot infer that sarcopenia is related to poor prognosis in patients with acute coronary syndromes without further high-quality data that specifically define sarcopenia according to formally accepted criteria.

#### 5.3.2. Coronary Artery Disease (CAD)

A recent meta-analysis by Li et al., which included 5 studies with a total of 783,626 predominantly elderly patients, most of which defined sarcopenia using the Asian Working Group on Sarcopenia (AWGS) criteria, found no association between sarcopenia and the risk of myocardial infarction [69]. However, indirect evidence that sarcopenia may be associated with atherosclerotic processes does exist. For example, a Korean study of predominantly middle-aged, apparently healthy adults (N = 31,108) found that skeletal muscle index (SMI) was associated in a dose-dependent manner with the coronary artery calcification (CAC) score, even after adjusting for multiple confounders [70]. Similar findings, plus an association of SMI with CAC progression, were reported some years later in a Canadian study of 19,728 middle-aged adults [71].

#### 5.3.3. Peripheral Arterial Disease (PAD)

Peripheral arterial disease (PAD) leads to reduced blood flow to lower limbs, restricting oxygen and nutrient supply, and potentially promoting a catabolic muscle state [72]. On the opposite end, patients with PAD often experience reduced physical activity or limited mobility due to discomfort or pain, which can further exacerbate sarcopenia by accelerating the loss of muscle mass and strength. Indeed, a small retrospective study by Takino et al. of 105 patients hinted at sarcopenia (using AWGS definition) being associated with decreased step count following endovascular treatment of PAD [73]. A low psoas muscle area index (PMI) was also associated with a 17.9% increased risk of amputation and a 20.8% increased risk of death at the 1-year follow-up of 190 patients with critical limb ischemia (CLI) [74]. The above, while not sufficient to elucidate the link between the two conditions, may point toward an unfavorable prognosis of PAD patients with sarcopenia.

Additionally, another small retrospective analysis of 101 patients with PAD showed ankle-branchial-index (ABI), a marker of PAD, to be a significant factor in predicting low muscle mass measured via bioimpedance analysis (OR 0.02, *p* = 0.027) [75]. However, whether this reflected regional muscle wasting from a reduction in blood supply or global muscle wasting cannot be inferred.

### 5.4. Atrial Fibrillation (AF)

Evidence has recently emerged that sarcopenia is a potential risk factor for the development of atrial fibrillation (AF). A prospective cohort trial by Tang et al. of 384,433 Caucasian patients with a mean age of 58 years, followed over a median of 12.56 years, revealed an association between the presence of sarcopenia and the incidence of AF [76]. In this study, sarcopenia was defined based on the EWGSOP2 consensus criteria. After adjusting for multiple factors and comorbidities, patients with probable sarcopenia (decreased hand-grip strength) were 8% (95% CI: 3–14%) more likely to develop AF, whereas those with confirmed sarcopenia (low muscle mass plus low strength) had a 61% (95% CI: 23–112%) higher likelihood of developing AF. Low hand-grip strength and low muscle mass by themselves had hazard ratios of 1.09 (95% CI: 1.04–1.15) and 1.21 (95% CI: 1.09–1.33), respectively. Subgroup analysis revealed that the effect of confirmed sarcopenia was consistent across all groups, with a higher risk observed in women, individuals under 65 years of age, and those with valvular heart disease. This study provides a robust conclusion, addressing what a previous study of 2225 participants from a Korean population failed to demonstrate [77]. Specifically, while Shim et al. found an association between sarcopenia and AF on cross-sectional analysis (adjusted OR = 2.13, 95% CI: 1.24–3.65), they were unable to demonstrate a longitudinal effect over 2 years of follow-up. Perhaps, the smaller sample, the older population (mean age 75.5), the shorter follow-up, or even the different population ethnicity contributed to this. It should also be noted that Shim et al. used the AWGS criteria to define sarcopenia.

The presence of sarcopenia could also have therapeutic implications for the anticoagulation management of patients with atrial fibrillation (AF). As muscle mass declines, the proportional concentration of hydrophilic DOAC drugs becomes lower in muscle and higher in plasma. Based on a small study, both peak and trough drug concentrations of apixaban and rivaroxaban seem to increase when appendicular lean mass decreases [78]. Consideration of sarcopenia in this context could help avoid bleeding events in those patients.

## 6. Treatment Approaches

The management of sarcopenia requires a holistic, multimodal approach targeting muscle mass preservation, functional improvement, and overall metabolic health [79]. Exercise is the cornerstone intervention, with resistance training (RT) demonstrating the most significant benefits in improving muscle mass, strength, and function. A regimen of RT performed 2–3 times weekly, focusing on large muscle groups with progressive overload, is recommended [80]. Aerobic exercise complements RT by enhancing cardiovascular fitness and reducing fat mass, indirectly improving muscle quality. Combined training approaches may yield superior outcomes [81].

Nutritional optimization is critical in sarcopenia management. Adequate protein intake (1.2–1.6 g/kg/day) is essential for muscle protein synthesis, with leucine-rich sources such as dairy, eggs, and lean meats requiring special attention. Supplementation with essential amino acids or whey protein may benefit individuals with suboptimal dietary intake. Vitamin D supplementation is necessary for those with deficiencies, as it supports muscle health and reduces fall risk. Iron supplementation should be considered for individuals with anemia, particularly if linked to sarcopenia-related fatigue or reduced exercise capacity. Omega-3 fatty acids may also play a supportive role in reducing inflammation and preserving muscle mass [81,82].

Hormonal therapies, particularly testosterone replacement in men with hypogonadism, have shown promise in increasing muscle mass and strength. However, their use must be carefully weighed against potential risks and contraindications. Growth hormone and selective androgen receptor modulators (SARMs) remain under investigation. Adequate management of comorbidities such as hypothyroidism and insulin resistance is equally important [83,84,85].

Non-pharmacologic interventions such as physiotherapy and balance training are crucial for improving physical function and preventing falls. Sleep optimization and stress reduction strategies, including cognitive-behavioral therapy and mindfulness, support recovery and overall metabolic balance. An individualized, patient-centered approach integrating exercise, nutrition, and lifestyle interventions, supported by pharmacologic treatments when necessary, is paramount in addressing sarcopenia effectively [81].

Emerging anti-aging strategies are under investigation for their potential to address sarcopenia, particularly by targeting the mechanisms underlying inflammaging, metainflammation, and muscle mass regulation. Key approaches include the use of NAD+ precursors, such as nicotinamide riboside (NR) and nicotinamide mononucleotide (NMN), to enhance mitochondrial function and reduce oxidative stress in muscle cells. Modulation of nutrient-sensing pathways, particularly through mTOR inhibitors such as rapamycin and AMPK activators such as metformin, has shown promise in mitigating muscle catabolism and promoting protein synthesis. Senolytic agents targeting pro-inflammatory senescent cells and interventions enhancing autophagy to restore cellular homeostasis may further counteract muscle degeneration. These strategies, when combined with personalized approaches integrating multi-omics data, hold the potential to reduce sarcopenia progression, improve muscle mass, and address the chronic inflammatory state characteristic of aging muscles. Further validation in clinical settings is essential to optimize these interventions for patient-specific outcomes [22].

## 7. Gaps in Evidence and Future Research Directions

The relationship between sarcopenia and cardiovascular diseases in the elderly remains a complex and underexplored area, leaving significant gaps in evidence. While sarcopenia’s association with HF, aortic stenosis, and ASCVD is increasingly recognized, the underlying mechanisms linking these conditions are not fully elucidated. For instance, the interplay between sarcopenia and the chronic low-grade inflammation characteristic of HF and ASCVD, known as “inflammaging”, warrants further investigation. The specific contributions of inflammatory cytokines, hormonal alterations, and metabolic dysregulation to muscle wasting in these settings need more robust data.

Moreover, heterogeneity in diagnostic criteria for sarcopenia, particularly in the context of cardiovascular diseases, poses a challenge. Studies on HF demonstrate variability in sarcopenia prevalence, influenced by differences in methods used to assess muscle mass and function. Similarly, in AS and TAVI populations, reliance on imaging-based assessments of muscle mass without standardized criteria contributes to inconsistent findings. The lack of studies employing comprehensive diagnostic tools, such as dual-energy X-ray absorptiometry (DXA) combined with functional assessments, limits the comparability and generalizability of results.

From a prognostic perspective, the bidirectional relationship between sarcopenia and HF highlights the need for longitudinal studies to determine causality. While sarcopenia exacerbates HF symptoms and contributes to poor outcomes, HF-related factors such as physical inactivity, oxidative stress, and malnutrition also promote sarcopenia. Understanding the temporal sequence and cumulative impact of these factors is critical for targeted interventions (correlation vs. causation).

Additionally, the role of advanced interventions, such as mTOR inhibitors, AMPK activators, and anti-inflammatory therapies, in modulating sarcopenia within cardiovascular populations remains underexplored. The potential of prehabilitation strategies, including nutritional supplementation and exercise regimens, to reverse sarcopenia prior to procedures such as TAVI is promising but requires validation in large-scale clinical trials.

Future research should prioritize the integration of multi-omics technologies and machine learning to uncover biomarkers that predict sarcopenia progression and its cardiovascular implications. Multicenter studies with standardized definitions and diagnostic protocols are necessary to address the current heterogeneity in data. Furthermore, interventions targeting the shared pathophysiological pathways of sarcopenia and cardiovascular diseases, such as inflammaging and metabolic dysregulation, should be a key focus. These efforts will pave the way for more effective and personalized strategies to mitigate the burden of sarcopenia in elderly cardiovascular patients.

## 8. Limitations

One major limitation of this review is the breadth of the subject matter and the inherent complexity and variety of pathophysiological mechanisms involved. Consequently, the selection of studies was guided by the authors’ judgment of relevance, which may have led to the exclusion of other potentially important work. Additionally, this review is neither a systematic review nor a meta-analysis, in part due to the large extent and the notable heterogeneity of the available literature—largely stemming from differing definitions and cutoff criteria for sarcopenia. While an evidence hierarchy was followed to evaluate and include studies, certain topics are supported only by limited data from small or observational studies, potentially constraining the generalizability of the conclusions. Furthermore, there is a potential for publication bias, as this review primarily included studies published in English, which may have excluded relevant findings from non-English sources.

## 9. Conclusions

Sarcopenia is an increasingly recognized condition in cardiogeriatrics, characterized by age-related declines in muscle mass, strength, and function, with significant implications for the elderly population, particularly those with cardiovascular comorbidities. The bidirectional relationship between sarcopenia and diseases such as heart failure, aortic stenosis, and atherosclerotic cardiovascular disease underscores the critical need for integrated management approaches. While diagnostic advancements have improved detection, heterogeneity in criteria continues to pose challenges. Emerging therapies targeting pathways such as mTOR, NAD+ metabolism, and inflammatory cytokines offer promising avenues but require further validation.

The integration of personalized strategies combining exercise, nutritional optimization, and novel anti-aging interventions represents a pivotal step forward. Additionally, leveraging machine learning and multi-omics approaches holds the potential to unravel biomarkers and therapeutic targets, enabling tailored interventions. Addressing these gaps through robust, multicenter research and standardized protocols will be essential to mitigate sarcopenia’s impact and improve health outcomes in aging cardiovascular populations. Through these efforts, the field of cardiogeriatrics can advance toward a more effective and comprehensive approach to managing sarcopenia and its associated risks.

## Figures and Tables

**Table 1:** Sarcopenia definitions, screening tools, and associated cutoff points.

Definition	Screening Tool	Muscle Mass	Muscle Strength	Physical Performance	Diagnostic Categories
EWGSOP2 (2019 revised consensus on the 2018 definition)	SARC-F ≥ 4 points	DXA or BIA	Grip Strength: <27 kg for men, <16 kg for women Chair Stand: >15 s for 5 rises	Gait Speed: ≤0.8 m/s SPPB: ≤8 points TUG: ≥20 s 400 m walk test: non-completion or ≥6 min to complete	Probable Sarcopenia: ↓ strength Confirmed Sarcopenia: ↓ strength combined with ↓ muscle quantity/quality Severe Sarcopenia: ↓ strength, ↓ muscle quantity/quality, and ↓ physical performance
		ASM: <20 kg for men, <15 kg for women ASM/h^2^: <7 kg/m^2^ for men, <5.5 kg/m^2^ for women			
AWGS (2019 consensus update on the 2014 definition)	CC: <34 cm in men, <33 cm in women SARC-F: ≥4 points SARC-CalF: ≥11 points	DXA	Grip Strength: <28 kg for men, <18 kg for women.	Gait Speed: <1 m/s SPPB: ≤9 points Five chair rises: ≥12 s	Possible sarcopenia: ↓ strength (primary care or community settings only) Sarcopenia: ↓ muscle mass plus either ↓ strength or ↓ physical performance Severe sarcopenia: ↓ muscle mass, ↓ strength, and ↓ physical performance
		ASM/h^2^: <7 kg/m^2^ for men, <5.4 kg/m^2^ for women BIA			
		ASM/h^2^: <7 kg/m^2^ for men, <5.7 kg/m^2^ for women			

ASM = Appendicular Skeletal Mass; CC = Calf Circumference; DXA = Dual-energy X-ray absorptiometry; BIA = Bioelectrical Impedance Analysis; SPPB = Short Physical Performance Battery; TUG = Timed Up-and-Go; ↓ (decreased).
